# Supplementary material for: Tailored Water-Soluble Covalent Organic Cages for Encapsulation of Pyrene and Information Encryption
Source: Int J Mol Sci. 2023 Dec 16;24(24):17541. doi: 10.3390/ijms242417541 (PMC10743434; doi:10.3390/ijms242417541)
Supplement: Supplementary file 1 [file ijms-24-17541-s001.zip › ijms-2715949-supplementary.pdf]

Supporting Information

1 General Procedures

Molecular Modeling. All the density-functional theory (DFT) computations were performed using the Gaussian 09 Software Package via the semiempirical PM6 method. The geometry optimization was conducted using the Berny method until the maximum force was below 0.00045 Ha/Bohr, and the maximum displacement was below 0.0018 Bohr.

^{1}H NMR Titration Experiments. All the titration experiments were performed in polar CD_3OD-d_6 solvent at room temperature. A solution of host **C** was prepared in an NMR tube. Next, the host solution was titrated with aliquots of a concentrated solution of guest. The host's proton signal was plotted as a function of the [G]/[H] molar ratio after each addition of the guest aliquot. Binding constants were obtained from nonlinear least-squares fitting of those titration curves using the Bindfit program reported by Thordarson's group.

2 Characterizations of compound 6 and cage C

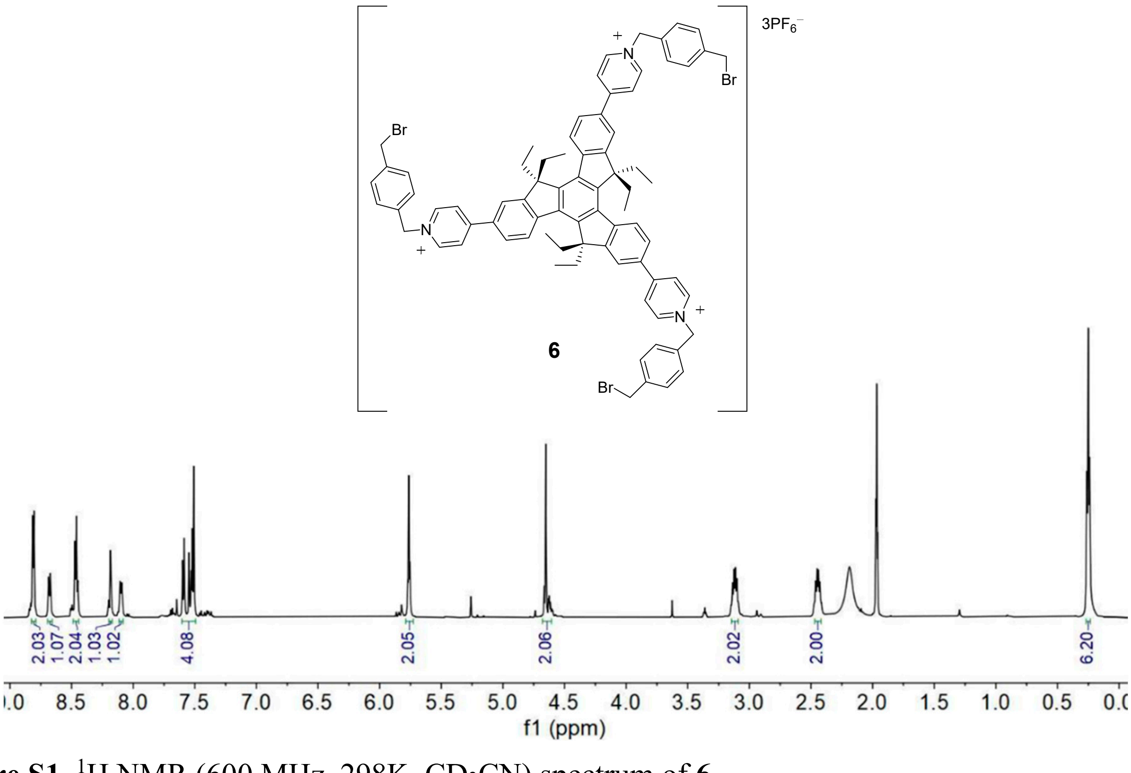

Figure S1. ^{1}H NMR (600 MHz, 298K, CD_3CN) spectrum of **6**.

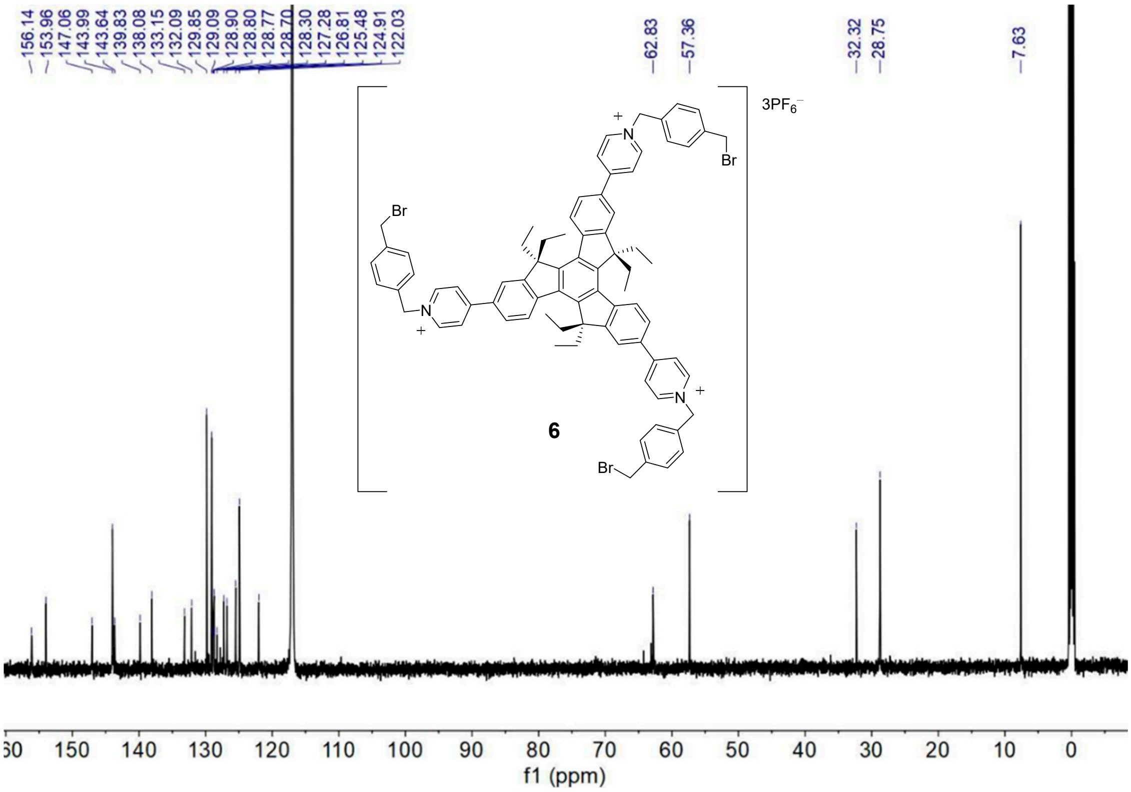

Figure S2. ^{13}C NMR (151 MHz, 298K, CD_3CN) spectrum of **6**.

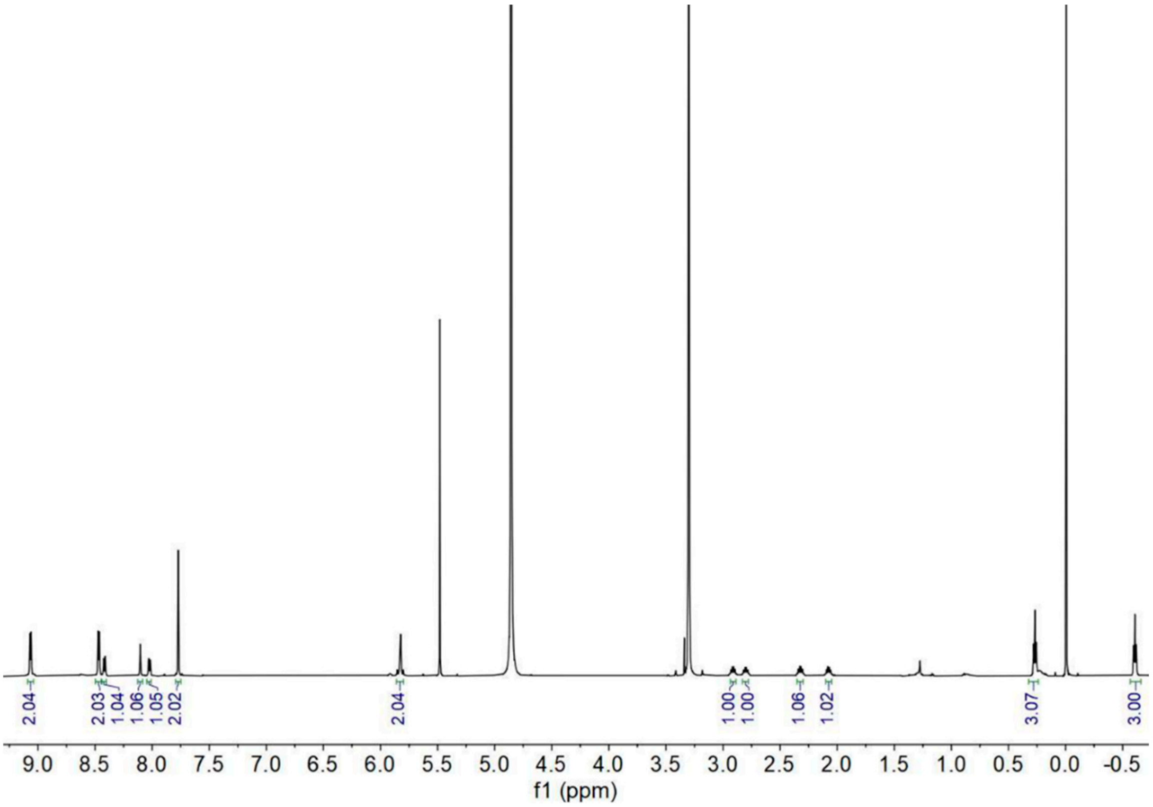

Figure S3. ^{1}H NMR (600 MHz, 298K, CD_3OD) spectrum of **C**.

3 ESI-MS data of covalent organic cage C

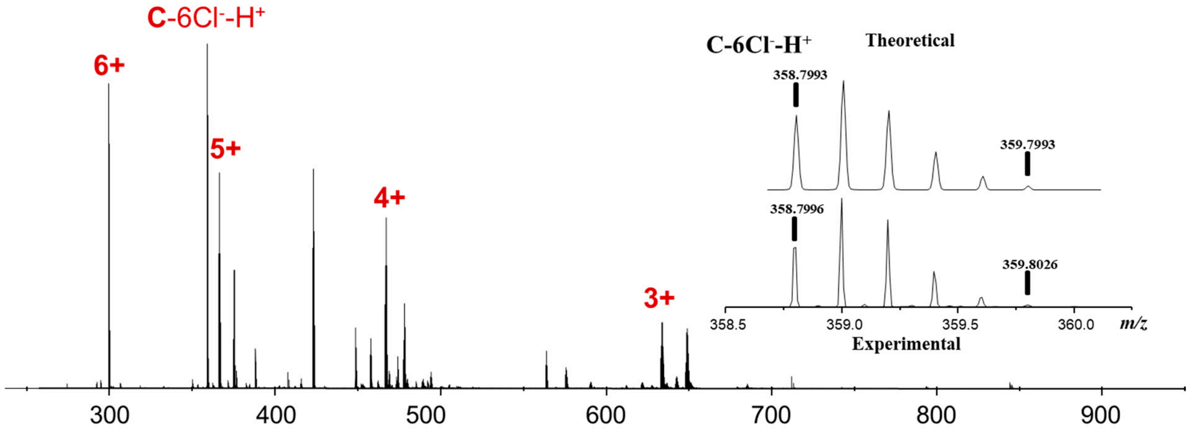

Figure S4. ESI-MS (D_2O) of **C**.

4 HRMS data of covalent organic cage C

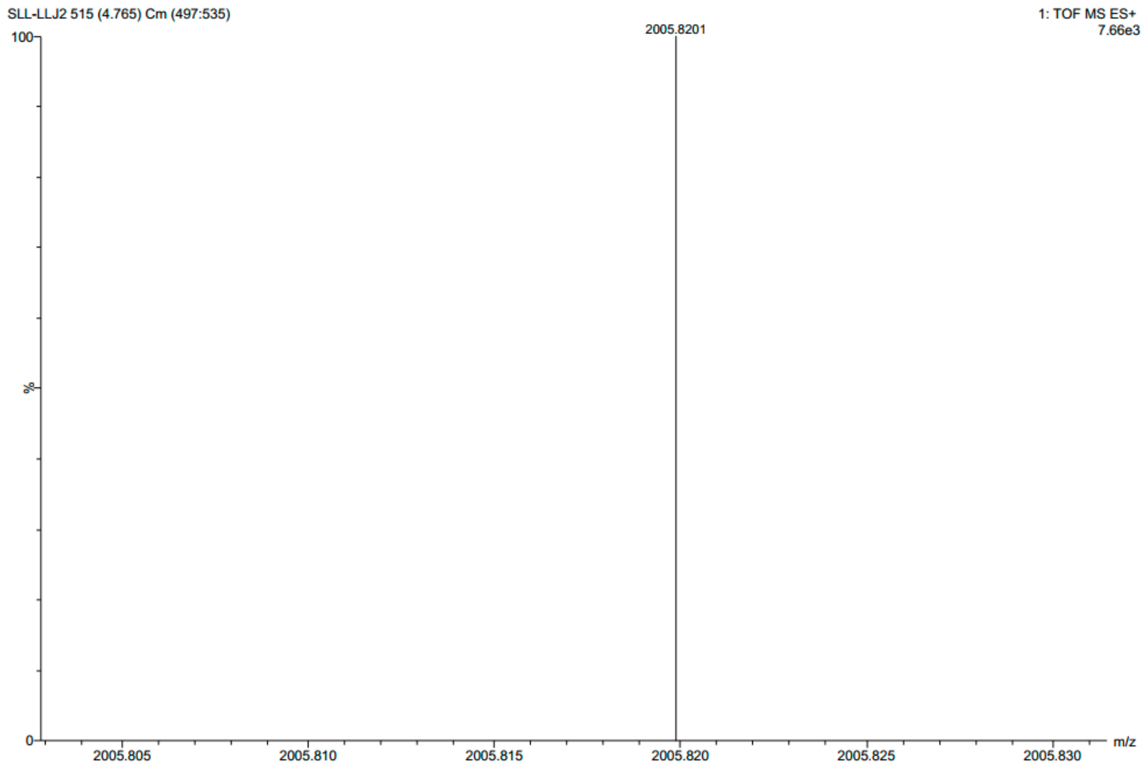

Figure S5. HRMS (D_2O) of **C**.

5 Optical property data of covalent organic cage C

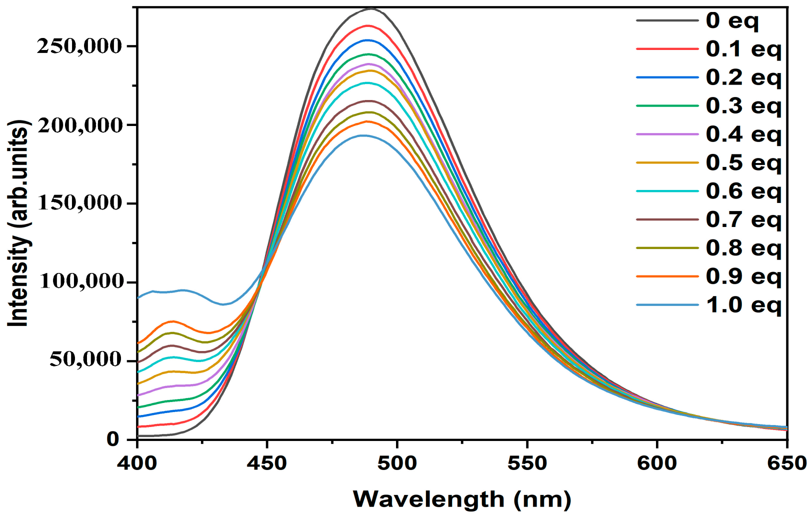

Figure S6. Fluorescence trend of covalent organic cage **C** with the addition of pyrene.

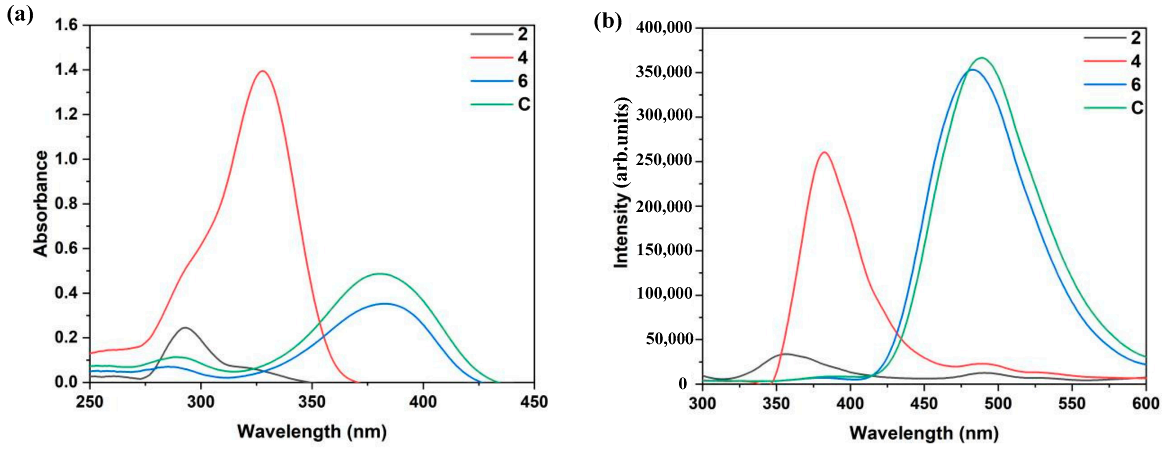

Figure S7. (a) UV-vis absorption spectra of cage **C** and **(b)** Fluorescence spectra of cage **C**.

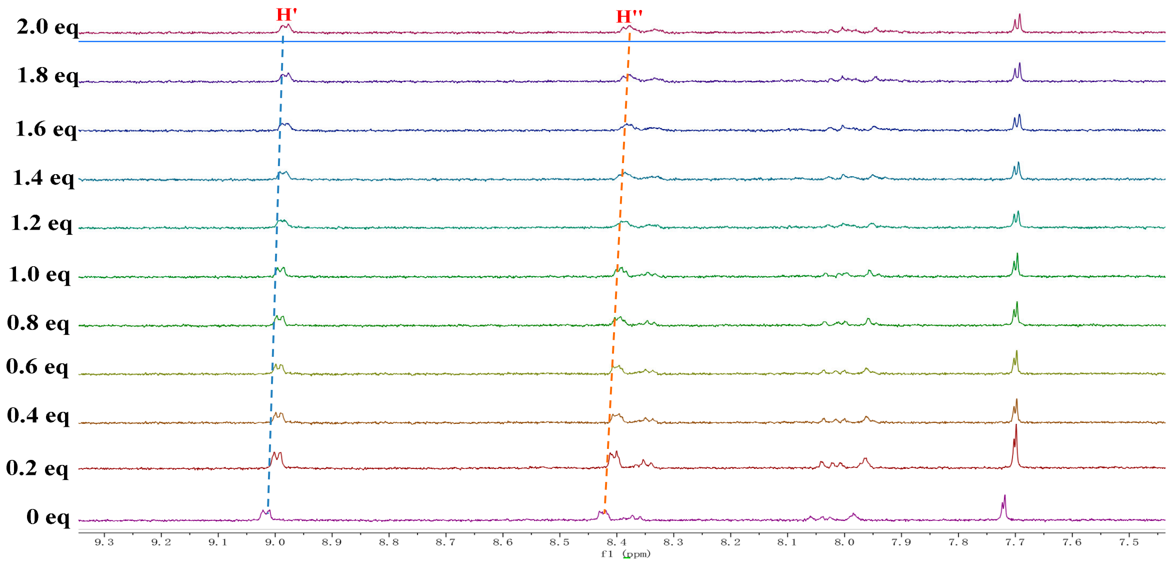

Figure S8. Partial ^{1}H NMR spectra for titration experiments of cage **C** with the addition of Pyrene in CD_3OD-d_6.

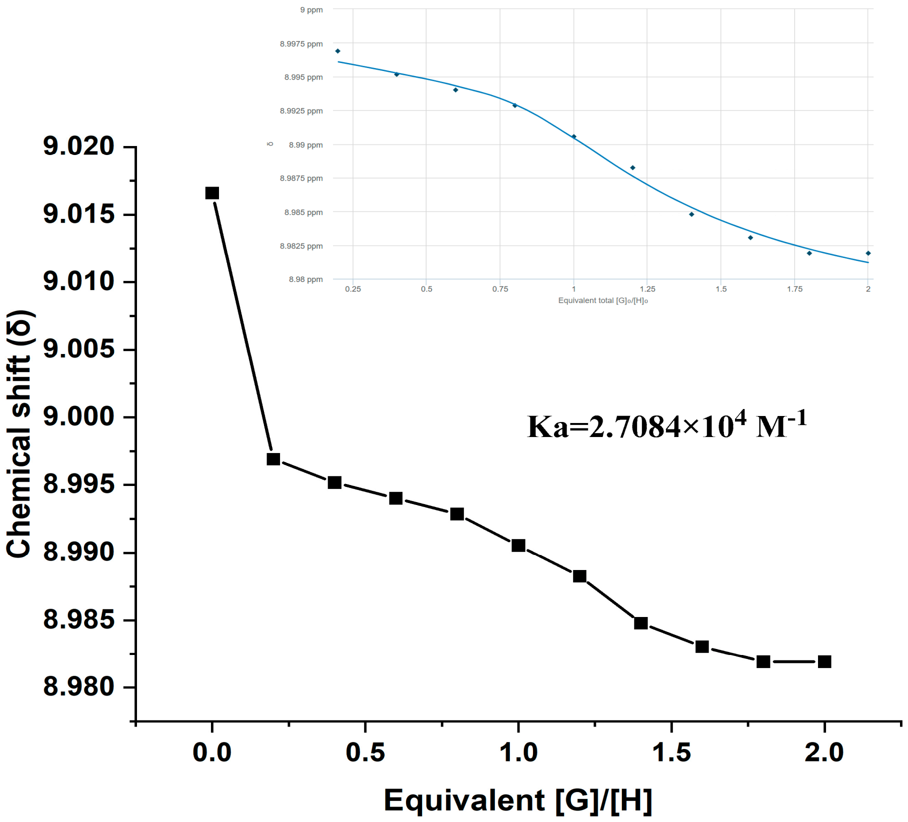

Figure S9. ^{1}H NMR titration curve of cage **C** from the chemical shifts (δ) of proton (H') versus equivalents of Pyrene plot. Inset: Fitting curve using the Bind-fit program (blue line).

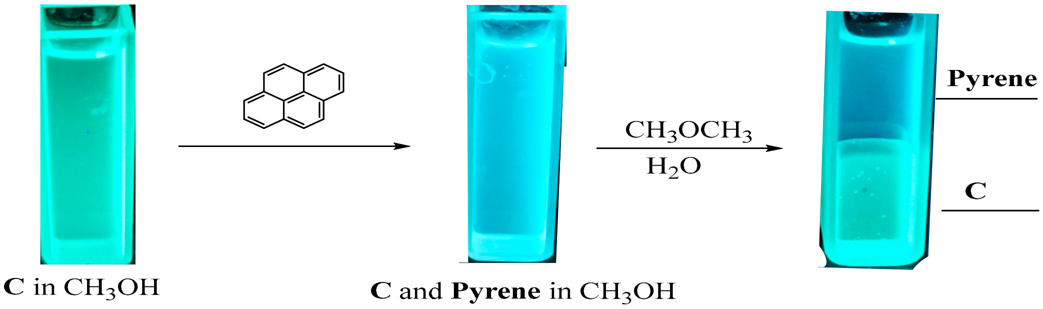

Figure S10. Interaction of covalent organic cage **C** with pyrene and its recycling.

6 References

1. Wu, H.; Wang, Y.; Song, B.; Wang, H.J.; Zhou, J.; Sun, Y.; Jones, L.O.; Liu, W.; Zhang, L.; Zhang, X., et al. A contorted nanographene shelter. *Nat Commun* **2021**, *12*, 5191, doi:10.1038/s41467-021-25255-6.

2. http://supramolecular.org
